# Maize *ZmGBSS1* Promotes Early Flowering and Enhances Drought Tolerance in *Arabidopsis*

**DOI:** 10.3390/plants15071093

**Published:** 2026-04-02

**Authors:** Ruirui Niu, Genlai Dong, Shizhan Chen, Wei Wang

**Affiliations:** 1 State Key Laboratory of High-Efficiency Production of Wheat-Maize Double Cropping, College of Life Sciences, Henan Agricultural University, Zhengzhou 450046, China; niuruirui@stu.henau.edu.cn; 2 Henan Plant Natural Products Development Engineering Research Center, Henan Academy of Sciences, Zhengzhou 450002, China; donggenlai@hnas.ac.cn

**Keywords:** *ZmGBSS1*, amylose, flowering time, drought tolerance, *Arabidopsis thaliana*

## Abstract

Granule-bound starch synthase (GBSS) is primarily recognized for its role in amylose production and starch granule formation in plant plastids. While its biochemical function in storage organs has been well documented, its broader contribution to plant growth and stress adaptation remains less defined. To explore these aspects, the maize gene *ZmGBSS1* was ectopically expressed in *Arabidopsis thaliana* and its physiological effects were examined. Subcellular localization assays confirmed that *ZmGBSS1* is specifically localized to chloroplasts. Phenotypic analysis of transgenic lines revealed that overexpression of *ZmGBSS1* significantly altered early seedling development, promoted root elongation, and accelerated flowering, with flowering occurring approximately four days earlier than in wild-type plants. Changes in development were accompanied by increased starch accumulation, elevated amylose levels, and a higher abundance of enlarged starch granules within chloroplasts. Under drought and PEG-induced osmotic stress, transgenic plants maintained improved growth performance and recovery capacity, together with greater proline accumulation and chlorophyll retention. These physiological advantages coincided with more rapid starch utilization and clear rises in transcripts for proline synthesis enzymes (AtP5CS1, AtP5CS2) and starch-degrading proteins (AtBAM1, AtBAM3, AtDPE1). Collectively, these findings suggest that *ZmGBSS1* not only regulates starch biosynthesis but also plays a crucial role in coordinating plant development and drought stress responses, highlighting its potential for improving stress tolerance through metabolic regulation.

## 1. Introduction

Starch is the principal non-structural carbohydrate in plants, acting both as a carbon reserve for energy storage and as a dynamic carbon source supporting growth and metabolism, thereby playing essential roles in plant development and stress adaptation [1,2]. It is composed of two glucose polymers, amylose and amylopectin, which are synthesized and organized into semi-crystalline granules within plastids [3,4,5]. Starch formation depends on the coordinated action of multiple plastid-localized enzymes, including ADP glucose pyrophosphorylase (AGPase), soluble starch synthases (SSs), starch branching enzymes (SBEs), debranching enzymes (DBEs), and granule-bound starch synthase (GBSS) [3,6,7]. Among these enzymes, GBSS catalyzes the elongation of predominantly linear glucan chains and is a major determinant of amylose accumulation, starch granule structure, and physicochemical characteristics [8,9,10].

Although starch metabolism has traditionally been investigated in storage organs, increasing evidence indicates that its functions extend to vegetative growth and developmental transitions [11,12,13]. In *Arabidopsis*, the daily turnover of transitory starch is crucial for maintaining carbon balance, which in turn affects vegetative growth and the timing of flowering. This regulation occurs through sugar signaling pathways that control key integrators such as FLOWERING LOCUS T (FT) and SUPPRESSOR OF OVEREXPRESSION OF CONSTANS 1 (SOC1), with disruptions in starch degradation or synthesis altering these processes [14,15,16]. Similar connections between starch dynamics and reproductive development have been observed in tomato and cereal crops [17,18,19,20,21,22]. Collectively, these findings demonstrate that starch metabolism is not merely associated with energy storage but is intricately integrated with developmental regulation across diverse plant species.

Drought stress is a major factor limiting crop productivity worldwide. It triggers extensive reprogramming of carbohydrate metabolism, often resulting in soluble sugar accumulation and dynamic changes in starch synthesis or degradation to support osmotic adjustment and cellular homeostasis [23,24]. Starch biosynthetic enzymes also contribute to stress-induced modulation of carbon allocation [25], while transcriptional networks integrate starch-derived metabolic signals with developmental processes such as flowering [26]. Starch metabolism plays a particularly important role in plant responses to abiotic stresses such as drought. Under water deficit, leaf transitory starch is actively degraded to produce soluble sugars and proline, helping maintain cellular integrity and osmotic balance [27,28,29]. In wheat, water limitation during early grain filling simultaneously activates both starch synthesis and degradation pathways, suggesting a dynamic reprogramming of carbon allocation to facilitate stress adaptation [30,31]. Similarly, in maize, drought-induced breakdown of transitory starch mediated by β-amylase *ZmBAM8* positively regulates stress tolerance [32]. Moreover, starch-deficient maize mutants exhibit nocturnal carbon starvation, excessive reactive oxygen species accumulation, restricted growth, and limited recovery following drought stress, highlighting the physiological importance of balanced starch production and remobilization [33]. Although GBSS-dependent amylose accumulation has been associated with enhanced drought responses in some systems [10,34,35], direct evidence linking specific GBSS isoforms to improved stress tolerance remains limited.

Previous studies have shown that starch metabolism can influence plant growth, development, and drought tolerance [30]. However, research on the role of maize *ZmGBSS1* in plant growth, development, and drought resistance has not yet been reported. To address this gap, we characterized maize granule-bound starch synthase 1 (*ZmGBSS1*) using heterologous expression in *Arabidopsis thaliana*, with particular emphasis on its effects on starch accumulation, developmental progression, and drought responsiveness. We hypothesized that ectopic expression of *ZmGBSS1* would enhance transitory starch accumulation in chloroplasts, accelerate the floral transition through improved carbon availability and sugar signaling, and improve drought tolerance by promoting more efficient starch remobilization and osmoprotectant production. The results indicate that elevated expression of *ZmGBSS1* leads to increased starch and amylose accumulation, accelerated floral transition, and enhanced tolerance to water deficit conditions. These findings highlight the broader physiological significance of GBSS proteins and suggest *ZmGBSS1* as a promising target for improving carbon use efficiency and abiotic stress adaptation in crops.

## 2. Results

### 2.1. Phylogenetic and Sequence Analysis of ZmGBSS1

**Figure 1:**
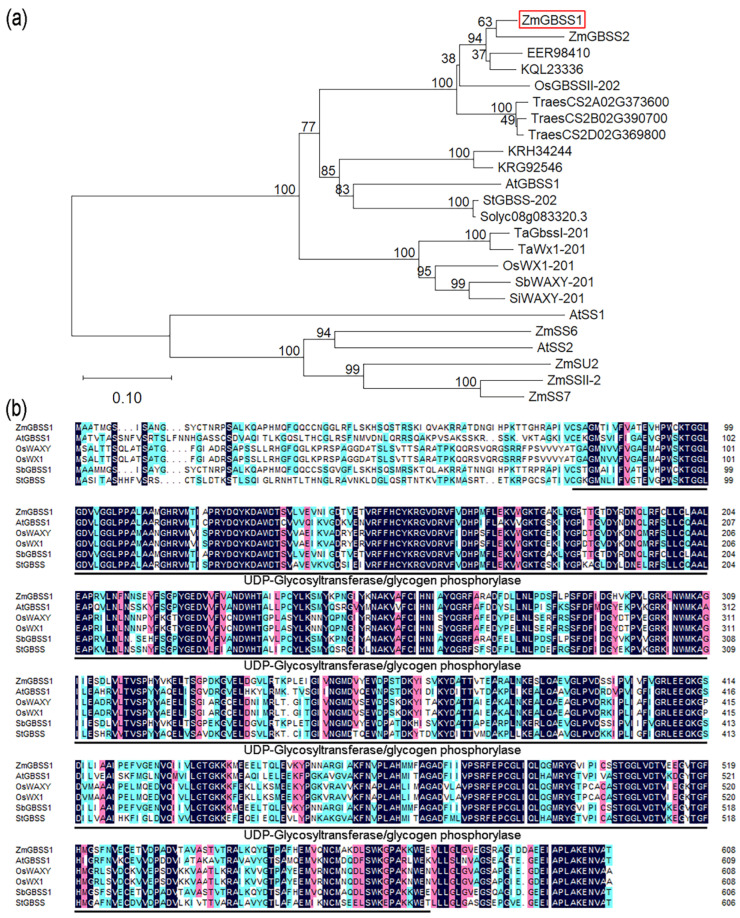
Phylogenetic tree and multiple sequence alignment analysis of ZmGBSS1 in maize. (**a**) Phylogenetic analysis of ZmGBSS1 protein. *Zea mays*: ZmGBSS1 (Zm00001eb305810), ZmGBSS2 (Zm00001eb000080), ZmSS6 (Zm00001eb222830), ZmSU2 (Zm00001eb279740), ZmSSII-2 (Zm00001eb254490), ZmSS7 (Zm00001eb194550); *Arabidopsis thaliana*: AtGBSS1 (AT1G32900), AtSS2 (AT3G01180), AtSS1 (AT5G24300); *Oryza sativa*: OsWX1-201 (Os06g0133000), OsGBSSII-202 (Os07g0412100); *Triticum aestivum*: TraesCS2A02G373600, TraesCS2B02G390700, TraesCS2D02G369800, TaGbssI-201 (TraesCS4A02G418200), TaWx1-201 (TraesCS7A02G070100); *Sorghum bicolor*: EER98410, SbWAXY-201 (SORBI_3010G022600); *Setaria italica*: SiWAXY-201 (KQL09140), KQL23336; *Glycine max*: KRH34244, KRG92546; *Solanum tuberosum*: StGBSS-202 (PGSC0003DMG400012111); *Solanum lycopersicum*: Solyc08g083320.3. ZmGBSS1 is highlighted in red. The red frame in panel (**a**) indicates the position of ZmGBSS1. (**b**) Multiple amino acid sequence alignment of ZmGBSS1 with GBSS orthologs from *Arabidopsis thaliana*, *Oryza sativa*, *Sorghum bicolor*, and *Solanum tuberosum*. Alignment was performed using DNAMAN 9.0 software, and domains were predicted via SMART (https://smart.embl.de/).

The *ZmGBSS1* gene sequence of maize was obtained from the MaizeGDB website. To explore its evolutionary position, we collected GBSS protein sequences from a range of representative plant species and built a phylogenetic tree. The analysis showed that ZmGBSS1 clustered within the monocot clade, together with GBSS homologs from *Oryza sativa*, *Triticum aestivum*, *Sorghum bicolor*, and *Setaria italica* (Figure 1a). This tight clustering points to strong evolutionary conservation of GBSS proteins across monocotyledonous species. Further comparison through multiple sequence alignment showed that ZmGBSS1 harbors a well-preserved glycosyltransferase domain typical of GBSS family members (Figure 1b). Several catalytic and substrate-binding regions were highly conserved across species, including homologs from *Arabidopsis thaliana*, *Oryza sativa*, *Sorghum bicolor* and *Solanum tuberosum*.

### 2.2. ZmGBSS1 Localizes to Chloroplasts

**Figure 2:**
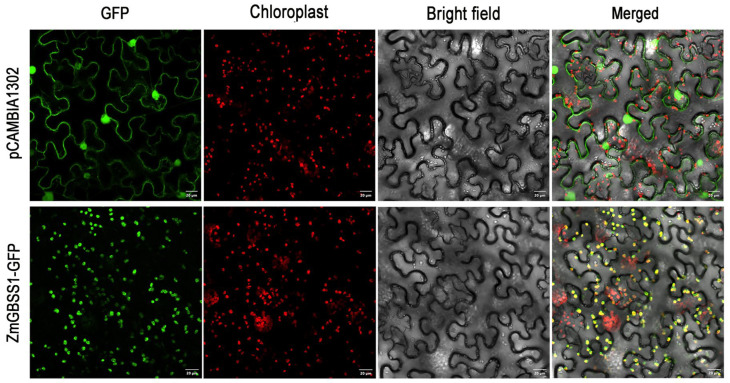
Subcellular localization of ZmGBSS1. GFP: Green fluorescent signal (excited at 488 nm); Chloroplast: red chloroplast autofluorescence (excited at 640 nm); Bright field: bright field image; Merge: merged images showing co-localization of GFP (green) and chloroplast autofluorescence (red). Scale bar = 20 μm.

To determine the functional localization of ZmGBSS1 protein in cells, we constructed and transiently expressed the ZmGBSS1-GFP fusion construct in *Nicotiana benthamiana*, and detected the GFP signal using confocal microscopy. As shown in Figure 2, the green fluorescence signal of ZmGBSS1-GFP was mainly detected in chloroplasts and highly overlapped with the chloroplast autofluorescence, while the control GFP protein driven by an empty vector was diffusely distributed throughout the cells. The merged images further confirm that ZmGBSS1 is specifically localized to chloroplasts, which is consistent with its functional prediction in starch biosynthesis (Figure 2).

### 2.3. Overexpression of ZmGBSS1 Affects Early Seedling Development in Arabidopsis

**Figure 3:**
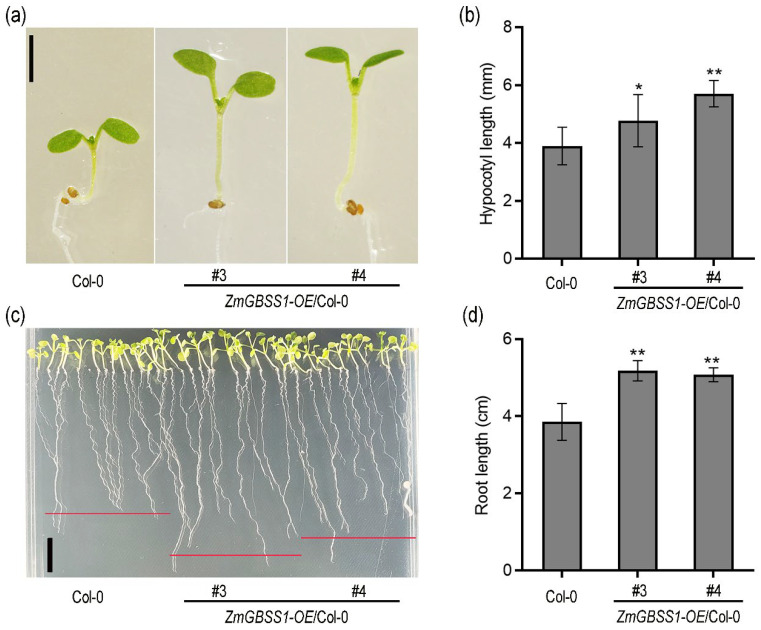
Effects of *ZmGBSS1* overexpression on early seedling growth in *Arabidopsis*. (**a**) Phenotypes of Col-0 and *ZmGBSS1* overexpression lines (#3 and #4) grown under continuous white light at 22 °C for 4 days. Bar = 2 mm. The red lines indicate the position of the root tips for primary root length measurement. (**b**) Quantification of hypocotyl length corresponding to (**a**). (**c**) Root growth of seedlings under the same conditions. Bar = 1 cm. (**d**) Quantification of primary root length corresponding to (**c**). The data are shown as means ± SD (*n* ≥ 10) (**b**,**d**). Statistical significance was determined using Student’s *t*-test (* *p* < 0.05, ** *p* < 0.01).

To explore the biological function of *ZmGBSS1*, we constructed a pJIM19-*ZmGBSS1* overexpression vector and introduced it into *Arabidopsis thaliana* (Col-0). PCR-based genotyping confirmed the successful acquisition of transgenic lines (Figure S1A). Two independent homozygous *ZmGBSS1* T3 lines (#3 and #4) exhibiting relatively higher transcript abundance were subsequently selected for detailed phenotypic characterization (Figure S1B). Under continuous white light, *ZmGBSS1* overexpressing seedlings exhibited significantly elongated hypocotyls compared to Col-0, with lines #3 and #4 showing 22.6% and 46.2% greater hypocotyl length, respectively (Figure 3a,b). In addition, primary root length was significantly enhanced in both transgenic lines, reaching approximately 1.32–1.35 times that of wild-type seedlings (Figure 3c,d). These results indicate that *ZmGBSS1* influences early seedling growth, suggesting a role in developmental regulation beyond starch storage.

### 2.4. ZmGBSS1 Promotes Early Flowering Under Long-Day Conditions

**Figure 4:**
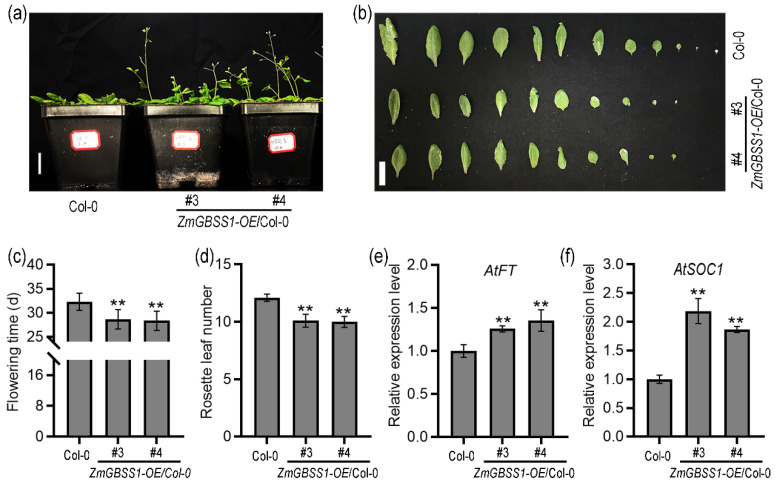
The adult plant phenotype of ZmGBSS1 transgenic Arabidopsis thaliana. (**a**) ZmGBSS1 transgenic Arabidopsis and Col-0 seedling morphology under long-day (LD, 16 h-light / 8 h-dark) conditions at 22 °C for 30 d. Bar = 1 cm. (**b**) Rosette leaf morphology at bolting stage. Bar = 2 cm. (**c**) Flowering time measured as days to bolting. (**d**) Number of rosette leaves at bolting. (**e**) qRT-PCR assays of relative expression of *AtFT*/*AtActin2* corresponding to (**a**). (**f**) qRT-PCR assays of relative expression of *AtSOC1*/*AtActin2* corresponding to (**a**). Data represent mean ± SD (*n* ≥ 10) (**c**,**d**); (*n* = 3) (**e**,**f**). Statistical significance was determined using Student’s *t*-test (** *p* < 0.01).

To determine whether *ZmGBSS1* affects the adult development stage of *Arabidopsis*, the growth status of *ZmGBSS1* overexpressing plants was observed under long-day (LD) conditions. Compared with Col-0, *ZmGBSS1* overexpressing plants flowered approximately four days earlier (Figure 4a,c). The fewer the number of rosette leaves in *Arabidopsis*, the earlier the flowering period [36,37]. In order to further confirm the effect of *ZmGBSS1* on flowering, statistics were conducted on the rosette leaves of *Arabidopsis thaliana*. The results showed that the number of rosette leaves at bolting was significantly reduced in transgenic lines, with an average decrease of two leaves relative to wild type (Figure 4b,d). *AtFT* and *AtSOC1* are positively regulating genes for flowering, and their increased expression levels promote early flowering in *Arabidopsis* [38,39,40]. To examine the molecular basis of this phenotype, the expression levels of flowering regulators were analyzed by qRT-PCR. Quantitative RT-PCR showed that the expression of *AtFT* was significantly upregulated in plants overexpressing *ZmGBSS1* (Figure 4e). Similarly, *AtSOC1* expression was markedly increased (Figure 4f).

To assess whether the enhanced starch accumulation comes at the cost of plant productivity, we examined seed-related traits at maturity under long-day conditions. Compared with wild-type Col-0, both *ZmGBSS1* overexpression lines produced significantly larger seeds, as indicated by increased projected seed area (Figure S2a,b). Consistent with this, thousand-seed weight was also markedly higher in the transgenic lines (Figure S2c). These findings indicate that *ZmGBSS1* improves carbon-use efficiency, leading to better seed filling without compromising overall plant performance.

### 2.5. ZmGBSS1 Overexpression Elevates Transitory Starch Levels in Arabidopsis

**Figure 5:**
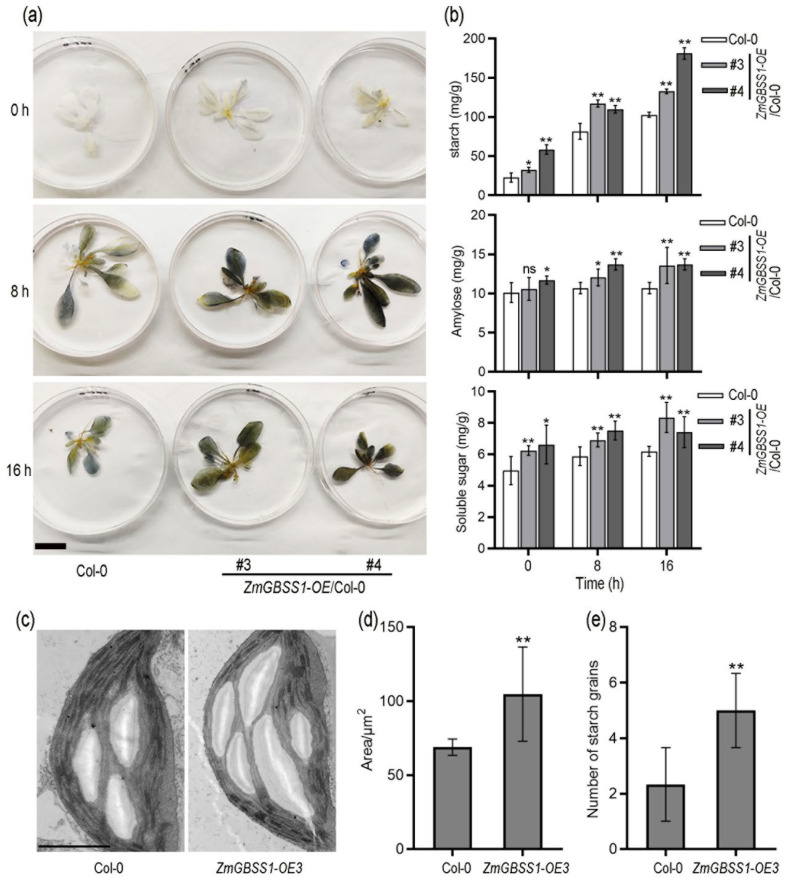
*ZmGBSS1* enhances starch accumulation in *Arabidopsis*. (**a**) Iodine (KI/I_2_) staining of leaves collected at 0, 8, and 16 h after the dark-to-light transition under long-day conditions. Bar = 1 cm. (**b**) Quantification of total starch, amylose, and soluble sugar contents using dried leaf material under the same conditions as in (**a**). (**c**) Transmission electron micrographs showing chloroplast starch granules in Col-0 and *ZmGBSS1* transgenic leaves. Bar = 2 μm. (**d**,**e**) Morphometric analysis of starch granule area and number per chloroplast section. Data represent mean ± SD (*n* ≥ 10) (**b**,**d**). Statistical analysis was performed using Student’s *t*-test (ns means no significant difference, * *p* < 0.05, ** *p* < 0.01).

GBSS is the key starch synthase for amylose synthesis, and by altering amylose content and starch structure, it indirectly influences plant growth [41,42]. To examine whether *ZmGBSS1* participates in regulating starch metabolism in photosynthetic tissues, we measured leaf starch accumulation in *ZmGBSS1* overexpressing *Arabidopsis* lines cultivated under long-day (LD) conditions. Seedlings grown under LD conditions were subjected to iodine staining (KI/I2) at 0, 8, and 16 h after light onset. Transgenic plants consistently displayed more intense blue-black staining than Col-0 controls, with the difference becoming particularly pronounced at the 8 h and 16 h time points (Figure 5a). Quantitative biochemical analysis of dried leaf samples further confirmed these observations. Under long-day conditions, the contents of total starch, amylose, and soluble sugars in *ZmGBSS1* overexpressing lines were consistently and significantly higher than those in wild-type plants at 0, 8, and 16 h following the dark-to-light transition (Figure 5b). Ultrastructural examination by transmission electron microscopy (TEM) further revealed that chloroplasts of transgenic leaves contained noticeably larger and more numerous starch granules than those of Col-0 (Figure 5c). Quantitative morphometric analysis verified these differences, showing significant increases in both average granule area and granule number per chloroplast section in the transgenic lines (Figure 5d,e).

To examine whether the introduced *ZmGBSS1* influences the expression of the endogenous *Arabidopsis* granule-bound starch synthase (*AtGBSS1*), we quantified *AtGBSS1* transcript levels in leaves, flowers, and developing seeds. *AtGBSS1* expression was significantly upregulated in all three tissues in both independent overexpression lines compared with the wild type (Figure S3). The presence of *ZmGBSS1* does not suppress, but rather upregulates, the native *AtGBSS1* gene.

### 2.6. ZmGBSS1 Overexpression Improves Drought Tolerance in Transgenic Arabidopsis

To evaluate whether *ZmGBSS1* is responsive to stress conditions in its native species, we analyzed its expression in maize seedlings subjected to PEG-induced osmotic stress. Maize inbred line B73 seedlings grown under long-day conditions for two weeks were subjected to PEG6000-induced osmotic stress. Leaf samples were collected at 0, 4, 8, 16, and 24 h after treatment, and ZmGBSS1 transcript levels were analyzed by qRT-PCR. The results revealed that *ZmGBSS1* transcript levels increased progressively during the early stages of stress. Expression rose steadily from 0 to 16 h, reaching a peak level approximately 13.9-fold higher than that at 0 h, before slightly declining at 24 h (Figure S4). These results indicate that ZmGBSS1 is responsive to osmotic stress in maize, suggesting that it may play a role in stress adaptation in its native context.

**Figure 6:**
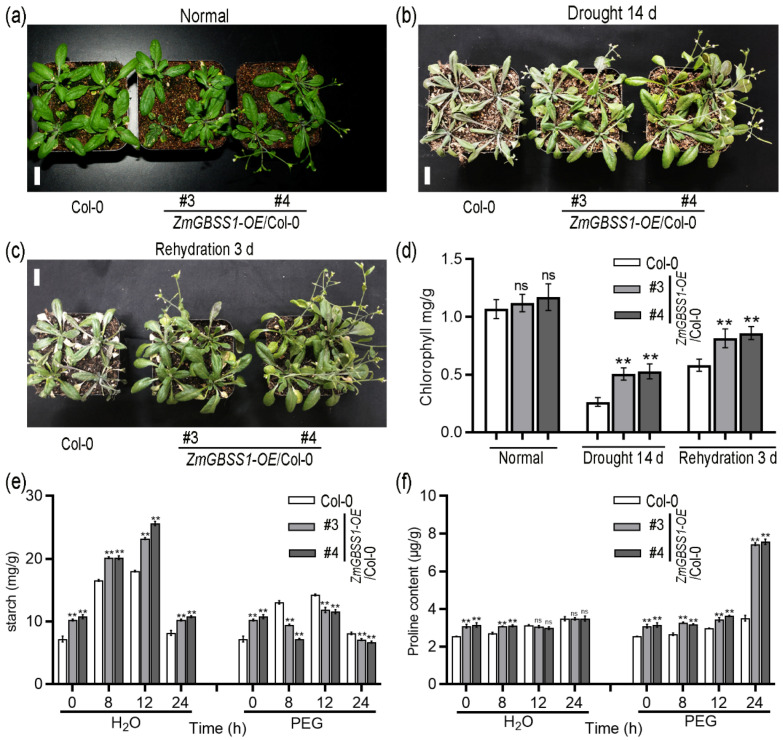
*ZmGBSS1* overexpression enhances drought tolerance in *Arabidopsis*. (**a**) Phenotypes of Col-0 and transgenic plants under normal watering conditions. (**b**) Phenotypes after 14 days of drought treatment. (**c**) Recovery phenotypes after 3 days of rewatering. Bars = 2 cm. (**d**) The chlorophyll content of the *Arabidopsis* leaves corresponding to (**a**–**c**). (**e**) Starch contents following PEG6000-induced osmotic stress at 0, 8, 12, and 24 h. (**f**) Proline content under PEG treatment at the indicated time points. For osmotic stress assays, 20-day-old plants were irrigated with 30% (*w*/*v*) PEG6000 solution. The data are shown as means ± SD (*n* ≥ 10) (**d**–**f**). Statistical significance was determined using Student’s *t*-test (ns means no significant difference, ** *p* < 0.01).

To assess whether *ZmGBSS1* influences drought tolerance in *Arabidopsis*, *ZmGBSS1* transgenic plants and Col-0 plants were subjected to drought stress and rehydration conditions. Under normal conditions, no obvious phenotypic differences were apparent between *ZmGBSS1* transgenic plants and wild-type Col-0 (Figure 6a). After 14 days without watering, *ZmGBSS1* transgenic plants displayed reduced wilting, maintained better turgor, and exhibited superior overall vigor compared to Col-0 (Figure 6b). Upon rewatering, the transgenic plants recovered more efficiently, displaying improved regrowth and healthier morphology (Figure 6c). Consistent with these observations, chlorophyll content remained markedly higher in drought-treated transgenic plants, reaching approximately 1.9–2.0 times that of Col-0 (Figure 6d).

The increase in soluble sugar and proline content can enhance the ability of plants to cope with drought stress [28,43]. We monitored starch, soluble sugars, and proline dynamics during PEG6000-induced osmotic stress at 0, 8, 12, and 24 h time points. Under normal watering conditions, *ZmGBSS1* transgenic plants already accumulated modestly higher starch and soluble sugar levels than Col-0, while proline showed only slight elevation at 0 and 8 h with no clear difference at 12 h and 24 h (Figure 6e,f and Figure S5). In response to PEG treatment, however, starch reserves in transgenic lines declined more sharply than in wild-type plants, becoming significantly lower at 8 h and 12 h. Concurrently, soluble sugars rose rapidly and remained elevated above Col-0 levels throughout the time course. Proline accumulation followed a similar pattern: transgenic plants exhibited higher proline at 0 h, 8 h, and 12 h, with the most pronounced difference at 24 h, where levels reached approximately 2.15-fold those of Col-0 (Figure 6e,f and Figure S5). These data show more rapid starch degradation and greater accumulation of soluble sugars and proline in the ZmGBSS1 overexpression lines under osmotic stress.

### 2.7. ZmGBSS1 Modulates Drought and Starch Metabolism Related Gene Expression Under PEG-Induced Stress

**Figure 7:**
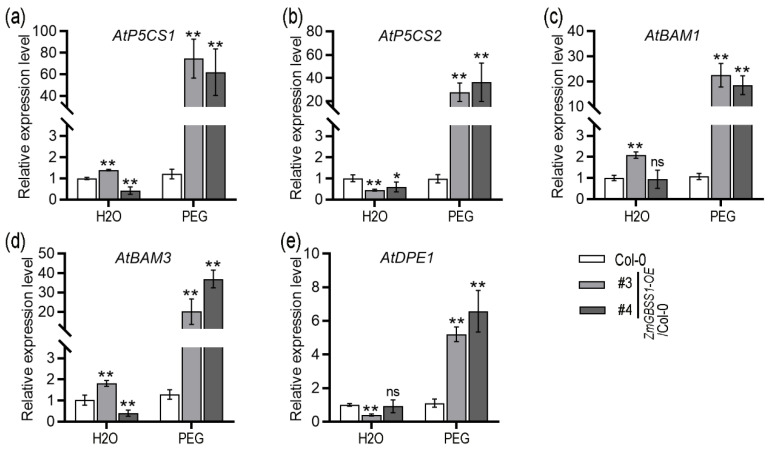
Expression analysis of drought- and starch metabolism-related genes under PEG-induced stress. Relative transcript levels determined by qRT-PCR after 8 h of PEG6000 treatment, normalized to Col-0. (**a**) *AtP5CS1* (AT2G39800). (**b**) *AtP5CS2* (AT3G55610). (**c**) *AtBAM1* (AT3G23920). (**d**) *AtBAM3* (AT4G17090). (**e**) *AtDPE1* (AT5G64860). The data are mean ± SD from three biological replicates. Statistical significance was determined using Student’s *t*-test (ns means no significant difference, * *p* < 0.05, ** *p* < 0.01).

In order to further investigate the molecular mechanism of drought resistance enhancement in *ZmGBSS1* transgenic plants, the expression levels of drought-related genes and starch metabolism genes under PEG-induced drought stress were analyzed. Leaf samples were collected from 20-day-old plants after 8 h of PEG treatment, and relative expression of five key genes was quantified by qRT-PCR: *AtP5CS1* and *AtP5CS2* (DELTA1-PYRROLINE-5-CARBOXYLATE SYNTHASE), *AtBAM1* and *AtBAM3* (β-amylase-mediated starch breakdown), and AtDPE1 (disproportionating enzyme involved in starch-to-sugar conversion) (Figure 7). Compared with wild-type Col-0, *ZmGBSS1* overexpressing lines showed dramatically higher expression of the proline biosynthetic genes *AtP5CS1* and *AtP5CS2*, which were induced on average by 56.5-fold and 32.5-fold, respectively. (Figure 7a,b). In addition, under drought stress, *AtBAM1* and *AtBAM3* involved in starch degradation, were also upregulated in transgenic lines (Figure 7c,d). Notably, *AtDPE1*, which facilitates the conversion of malto-oligosaccharides into soluble sugars during starch breakdown, showed strong upregulation in *ZmGBSS1* transgenic plants (Figure 7e). These transcriptional changes support the physiological observations and indicate that *ZmGBSS1* may enhance drought tolerance through coordinated regulation of starch metabolism and osmoprotectant biosynthesis.

## 3. Discussion

Granule-bound starch synthase (GBSS) catalyzes amylose formation and thereby determines starch composition and carbon partitioning in plants [42,44]. In this study, ZmGBSS1 was shown to localize to chloroplasts (Figure 2) and share high sequence conservation with GBSS homologs from other species, including a conserved UDP-glycosyltransferase domain (Figure 1). Overexpression of *ZmGBSS1* in *Arabidopsis* resulted in increased total starch and amylose contents, accompanied by enlarged and more abundant starch granules in chloroplasts, as confirmed by iodine staining, biochemical measurements, and transmission electron microscopy (Figure 5). Although direct enzymatic activity of GBSS was not assessed, the phenotypic changes observed here are commonly considered indicative of increased GBSS function during transitory starch metabolism [2,5]. Such observations are in good agreement with earlier studies showing that boosted GBSS activity elevates the amylose fraction and modifies granule morphology across different plant systems [10,45,46,47].

Seedlings overexpressing *ZmGBSS1* displayed elongated hypocotyls and primary roots and transitioned to flowering approximately four days earlier than wild-type plants under long-day conditions (Figure 3 and Figure 4). This phenotype is likely associated with changes in carbon metabolism and sugar signaling. In *Arabidopsis*, soluble sugars derived from starch turnover are not only essential metabolic substrates but also act as signaling molecules that influence developmental transitions, including the switch from vegetative to reproductive growth [48,49]. Accumulating evidence indicates that sugar-related pathways are integrated into the flowering regulatory network. These signals ultimately converge on a small number of key floral integrators, such as *FT* and *SOC1*, which coordinate the floral transition [50]. In addition, changes in sugar availability and transport have been shown to be closely associated with FT-dependent flowering regulation, further supporting a functional link between carbon status and flowering control [51]. In this context, the increased starch accumulation observed in *ZmGBSS1* overexpression plants may enhance the supply of soluble sugars through subsequent degradation, thereby altering cellular carbon status. Such changes may promote sugar signaling pathways that contribute to the upregulation of *AtFT* and *AtSOC1* (Figure 4e,f). Therefore, *ZmGBSS1* is more likely to influence flowering indirectly by modulating carbon allocation and sugar signaling rather than acting directly on flowering regulatory genes. Notably, overexpression of *ZmGBSS1* promoted early flowering in *Arabidopsis* while also enhancing grain yield. Under normal growth, the transgenic plants produced significantly larger seeds with higher thousand-seed weight (Figure S2), indicating improved carbon allocation to reproductive sinks without an obvious growth penalty.

Although starch and soluble sugars frequently show an inverse relationship, the *ZmGBSS1* overexpression lines accumulated higher levels of both under normal growth conditions (Figure 5b). The enlarged amylose-rich starch granules allow greater daytime carbon storage without strongly inhibiting photosynthesis, while a modestly accelerated turnover rate maintains elevated soluble sugar levels. Under osmotic stress, the transgenic plants break down starch more quickly (Figure 6e), resulting in higher soluble sugars and proline contents that support osmotic adjustment (Figure 6f and Figure S5). These results suggest that *ZmGBSS1* enhances overall carbon-use efficiency rather than simply shifting the balance between storage and export. A similar pattern of simultaneous increases in starch and soluble sugars has been observed when starch biosynthetic capacity is strengthened, for example, in *Arabidopsis* overexpressing the grape ADP-glucose pyrophosphorylase large subunit (*VaAPL1*) [52]. Collectively, these findings indicate that ZmGBSS1 optimizes diurnal carbon storage and release, thereby improving both vegetative growth and stress resilience.

A particularly striking phenotype was the improved drought performance of *ZmGBSS1*-overexpressing plants (Figure 6). These plants wilted less severely during water withholding, recovered more quickly after rewatering, and maintained higher chlorophyll levels compared to controls. This enhanced ability to withstand stress seems closely linked to the way *ZmGBSS1* affects osmotic adjustment, as shown by the greater buildup of proline and soluble sugars in the *ZmGBSS1* transgenic plants during drought (Figure 6e,f). Proline is a well-established osmoprotectant that accumulates in response to water deficit and protects cellular structures from dehydration-induced damage [53,54,55]. Additionally, the increase in starch reserves may serve as an energy source during drought, providing carbon for vital metabolic processes under water stress [33,56]. Such responses suggest that *ZmGBSS1* facilitates stress-induced starch remobilization, supplying substrates for osmoprotectant synthesis and osmotic adjustment. Recent studies have highlighted the complex role of starch metabolism in drought responses, not only as a source of carbon and osmoprotectants but also through its interaction with transcriptional networks that coordinate stress adaptation and development [48,49,50,51]. Gene expression data further support this idea. Under osmotic stress, *ZmGBSS1*-overexpressing plants showed strong upregulation of proline biosynthetic genes *AtP5CS1* and *AtP5CS2*, along with starch-degrading enzymes *AtBAM1* and *AtBAM3* (Figure 7). *AtP5CS1* and *AtP5CS2* are involved in proline biosynthesis, which is known to be critical for drought tolerance, while *AtBAM1* and *AtBAM3* are involved in starch degradation, which may provide an energy source during periods of stress [27,57,58,59]. The increased expression of these genes in *ZmGBSS1* overexpression plants suggests that *ZmGBSS1* acts via altered sugar signaling or other metabolic cues, rather than direct regulation (Figure 7).

A potential limitation of the present study is the use of heterologous expression in *Arabidopsis* rather than direct functional analysis in maize. Nevertheless, we found that *ZmGBSS1* is strongly and rapidly upregulated in maize leaves under PEG-induced osmotic stress, with transcript levels increasing up to 13.9-fold at 16 h (Figure S4). This native stress-responsive expression pattern is consistent with the improved drought tolerance and accelerated starch turnover observed in the *Arabidopsis* transgenic lines, suggesting that *ZmGBSS1* likely plays a similar role in regulating carbon metabolism and stress adaptation in maize. Future work will require direct validation in maize, such as CRISPR-based knockout or overexpression lines. Crosses with starch metabolism or flowering mutants would also help clarify whether the effects of *ZmGBSS1* are direct or indirect, and assess its potential value for crop improvement under drought conditions.

Overall, our findings indicate that *ZmGBSS1* plays a pivotal role in enhancing drought tolerance in *Arabidopsis* by promoting starch accumulation, regulating osmoprotectants like proline, and modulating the expression of drought-responsive genes. This work provides insights into the potential application of *ZmGBSS1* for improving stress tolerance in crops, particularly in environments subject to water scarcity.

## 4. Materials and Methods

### 4.1. Plant Materials and Growth Conditions

*Arabidopsis thaliana* seeds were surface-sterilized and subjected to stratification, as previously outlined [60]. The sterilized seeds were deposited onto half-strength Murashige and Skoog (½ MS) medium enriched with 1% (*w*/*v*) sucrose and 0.8% (*w*/*v*) agar for germination. For phenotypic analyses in adult plants, seedlings were subsequently transferred to soil. The plants were grown in a 22 °C light culture chamber with 16 h of light and 8 h of darkness, and the intensity of white light was approximately 70 μmol m^−2^ s^−1^.

### 4.2. Generation of ZmGBSS1 Transgenic Arabidopsis

The full-length coding sequence of *ZmGBSS1* (Zm00001eb305810) was amplified from maize leaf cDNA using gene-specific primers (Supplementary Table S1). The *ZmGBSS1* gene was cloned into the overexpression vector pJIM19. The pJIM19 vector uses the CaMV35S promoter to drive constitutive overexpression of *ZmGBSS1*. The pJIM19-*ZmGBSS1* recombinant construct was introduced into *Agrobacterium tumefaciens* strain GV3101 by electroporation. The Agrobacterium was transformed into *Arabidopsis thaliana* using the floral-dip method [61]. T1 seeds were screened on MS medium supplemented with 25 mg/L kanamycin. Resistant seedlings were self-pollinated, and homozygous T3 lines showing stable high expression of *ZmGBSS1* were identified through PCR and qRT-PCR. Two independent homozygous T3 lines (#3 and #4), which exhibited the highest levels of *ZmGBSS1* transcript accumulation, were selected for further phenotypic and molecular characterization (Figure S1).

### 4.3. Sequence Retrieval and Phylogenetic Analysis

Homologous sequences of GBSS proteins from various monocot and dicot species were sourced from EnsemblPlants. Using the MEGA 11.0.13 software, a phylogenetic tree of the GBSS protein based on the maximum likelihood method was constructed.

### 4.4. Subcellular Localization Assay

To determine subcellular localization of *ZmGBSS1*, the gene was fused in-frame with GFP in the pCAMBIA1302 vector, creating the pCAMBIA1302–*ZmGBSS1*–GFP construct. The recombinant plasmid was transformed into the *Agrobacterium tumefaciens*, and a suspension of Agrobacterium cells carrying the construct was injected into the abaxial surface of 30-day-old *Nicotiana benthamiana* leaves. After 24 h of incubation in darkness to allow transient expression, fluorescence was observed using a Leica TCS SP8 STED ONE confocal microscope (Leica Microsystems, Wetzlar, Germany). Primers for construct assembly are detailed in Supplementary Table S1.

### 4.5. Phenotypic Analysis

For seedling growth analysis, the sterilized seeds were grown vertically on ½ MS plates for 4 days in a growth environment of 22 °C under continuous white light. The lengths of the hypocotyls and primary roots of *Arabidopsis* were measured using ImageJ 1.54g software. At least 10 seedlings from each strain were analyzed.

Rosette leaf number at bolting was recorded at the onset of flowering. For mature plant phenotyping, plants were grown under long-day (LD) conditions for 30 days, and rosette leaf number, flowering time, and overall plant morphology were assessed. At least 10 plants per genotype were evaluated under long-day conditions.

### 4.6. Measurement of Starch, Amylose, Soluble Sugar Content, and Starch Staining

For iodine staining, leaves were harvested at 0, 8, and 16 h after the dark-to-light transition under long-day conditions. Chlorophyll was removed by boiling in 95% ethanol, and tissues were stained with Lugol’s iodine solution (KI/I_2_). Total starch content was determined using a commercial starch assay kit (Boxbio Science & Technology Co., Ltd., Beijing, China). Amylose content was quantified with an amylose quantification kit (Solarbio Science & Technology Co., Ltd., Beijing, China). Soluble sugar content was measured using a soluble sugar assay kit (Boxbio Science & Technology Co., Ltd., Beijing, China). All measurements were performed with at least three biological replicates.

### 4.7. Transmission Electron Microscopy (TEM)

TEM sample preparation and imaging were performed by Servicebio Biotechnology Co., Ltd. (Wuhan, China). Leaf samples were fixed in 2.5% glutaraldehyde, post-fixed in 1% osmium tetroxide, and subjected to dehydration through a graded ethanol series, followed by embedding in epoxy resin. Ultrathin sections were stained with uranyl acetate and lead citrate and observed under a transmission electron microscope. The number and area of starch granules per chloroplast were quantified using ImageJ software.

### 4.8. Drought Stress and PEG-Induced Osmotic Stress Treatments

*ZmGBSS1* transgenic *Arabidopsis* and the control Col-0 were grown in soil at 22 °C under white light for 20 days, followed by 14 days of water deprivation stress. The control plants received regular irrigation. Phenotypic changes were recorded under well-watered conditions, after the drought period, and following 3 days of rehydration. Leaf chlorophyll was extracted in 80% (*v*/*v*) acetone for spectrophotometric quantification.

Osmotic stress was imposed on 20-day-old plants with 30% (*w*/*v*) PEG6000 solution. Leaf samples were collected at 0, 8, 12, and 24 h for biochemical analysis, including proline concentration, starch, and soluble sugar content determination. Proline content was measured using a commercial detection kit (Solarbio Science & Technology Co., Ltd., Beijing, China). Starch and soluble sugar determinations followed the protocols outlined in Materials and Methods Section 4.6.

### 4.9. RNA Extraction and Quantitative Real-Time PCR (qRT-PCR)

Total RNA was extracted from leaf tissues with TransZol reagent (TransGen Biotech, Beijing, China). Reverse transcription to first-strand cDNA was conducted using the EasyScript^®^ One-Step gDNA Removal and cDNA Synthesis SuperMix (Transgen biotech, Beijing, China), adhering to the manufacturer’s instructions. qRT-PCR was conducted with THUNDERBIRD^®^ Next SYBR qPCR Mix (TOYOBO, Shanghai, China) using a LightCycler 480 II Real-Time PCR System (Roche, Basel, Switzerland). Transcript levels were normalized to the reference gene *ACT2*, and relative transcript levels were calculated using the 2^^–ΔΔCt^ method. Each experiment included three independent biological replicates. Quantitative primers for *AtFT*, *AtSOC1*, *AtP5CS1*, *AtP5CS2*, *AtBAM1*, *AtBAM3*, and *AtDPE1* were designed using Primer Premier 5.0 software. The sequences are listed in Supplementary Table S1.

For maize stress expression analysis, maize B73 seedlings grown under long-day conditions were irrigated with 20% (*w*/*v*) PEG6000 solution. Leaf samples were harvested at 0, 4, 8, 16, and 24 h after treatment. Total RNA extraction and qRT-PCR were performed as described above, using *ZmTublin* as the internal reference gene. Primers for *ZmGBSS1* are listed in Supplemental Table S1.

## 5. Conclusions

In conclusion, heterologous expression of *ZmGBSS1* in *Arabidopsis* leads to alterations in carbon distribution, resulting in increased starch accumulation, faster flowering, and improved drought tolerance. These findings extend the known function of *ZmGBSS1* beyond its role in amylose synthesis in storage tissues, linking transitory starch metabolism with both developmental timing and stress response pathways. Considering the pivotal role of starch dynamics in plant productivity under changing environmental conditions, *ZmGBSS1* emerges as a candidate gene worthy of further evaluation in maize and other cereals for potential improvement of drought tolerance.

## Data Availability

The data are contained within the article or Supplementary Materials.

## Supplementary Materials

The following supporting information can be downloaded at https://www.mdpi.com/article/10.3390/plants15071093/s1: Figure S1: Molecular identification of *ZmGBSS1* transgenic *Arabidopsis thaliana*; Figure S2. Seed size and thousand-seed weight of Col-0 and *ZmGBSS1* overexpression lines under long-day conditions; Figure S3. Expression of the endogenous *AtGBSS1* gene in leaves, flowers, and seeds of Col-0 and *ZmGBSS1* overexpression lines; Figure S4. Expression pattern of *ZmGBSS1* in maize B73 leaves under 20% PEG6000 osmotic stress; Figure S5: The content of soluble sugars induced by PEG6000; Table S1: Primers used in this study.
