# Supplementary material for: Maize ZmGBSS1 Promotes Early Flowering and Enhances Drought Tolerance in Arabidopsis
Source: Plants (Basel). 2026 Apr 2;15(7):1093. doi: 10.3390/plants15071093 (PMC13075131; doi:10.3390/plants15071093)
Supplement: Supplementary file 1 [file plants-15-01093-s001.zip › plants-4211138-supplementary.pdf]

Supplementary Material

Supplemental data the following materials are available in the online version of this article

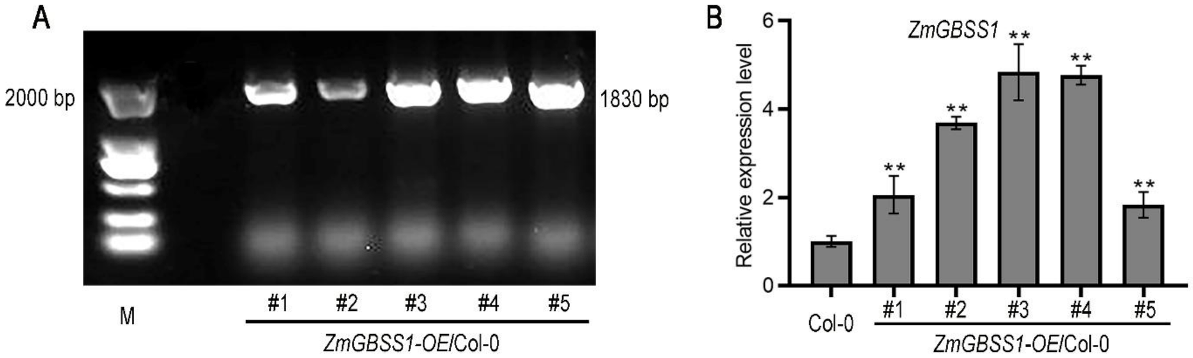

Supplemental Figure S1. Molecular identification of *ZmGBSS1* transgenic *Arabidopsis thaliana*. (**a**) PCR detection of the *ZmGBSS1* gene in transgenic *Arabidopsis thaliana*. In the figure: M represents the DNA marker DL 2000, the length of the target fragment is 1830 bp, #1-#5 represent the positive transgenic plants transformed with pJIM19-*ZmGBSS1*. (**b**) QRT-PCR verification of transgenic *Arabidopsis thaliana*. Asterisks mark significant differences between Col-0 with the *ZmGBSS1* transgenic lines according to Student's *t*-test (** $P < 0.01$). The experiment was performed using three biological replicates.

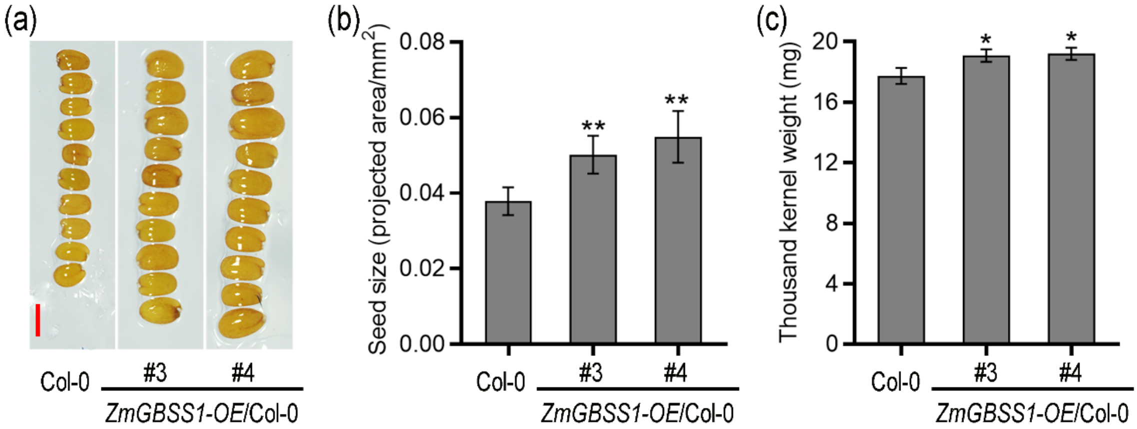

Supplemental Figure S2. Seed size and thousand-seed weight of Col-0 and *ZmGBSS1* overexpression lines under long-day conditions. (**a**) Representative images of mature seeds. Bar = 0.2 mm. (**b**) Projected seed area. (**c**) Thousand kernel weight. Data represent mean ± SD (n ≥ 20). Asterisks indicate significant differences from Col-0 (* $P < 0.05$, ** $P < 0.01$, Student's *t*-test).

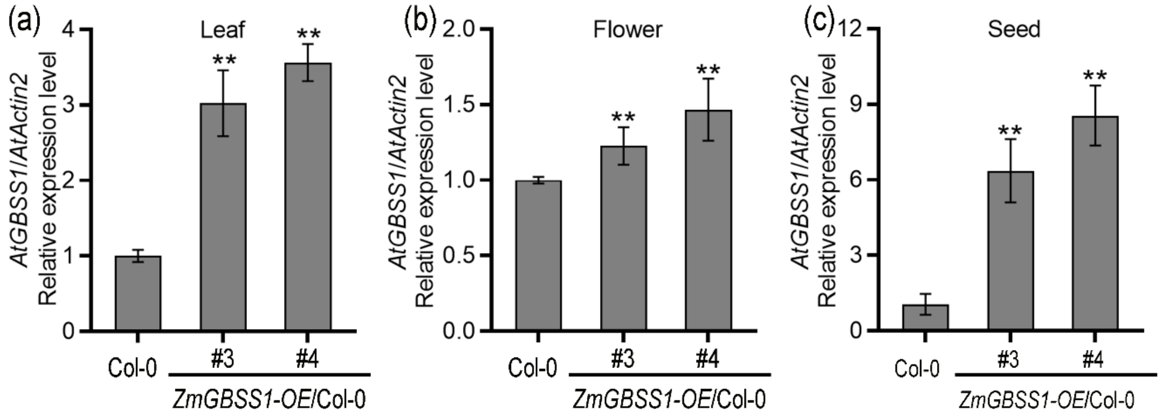

Supplemental Figure S3. Expression of the endogenous *AtGBSS1* gene in leaves, flowers, and seeds of Col-0 and *ZmGBSS1* overexpression lines. Transcript levels were determined by qRT-PCR and normalized to AtActin2. Asterisks indicate significant differences from Col-0 (** $P < 0.01$, Student's *t*-test). The experiment was performed using three biological replicates.

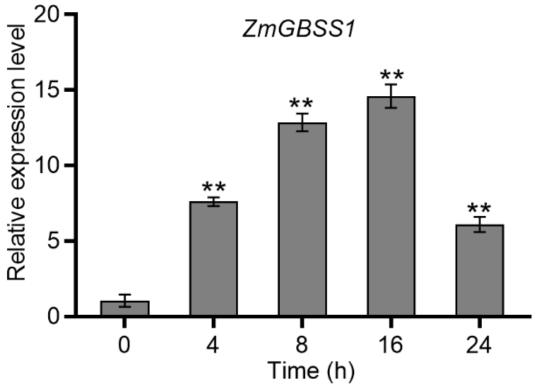

Supplemental Figure S4. Expression pattern of *ZmGBSS1* in maize B73 leaves under 20% PEG6000 osmotic stress. Maize inbred line B73 seedlings grown under long-day conditions for two weeks were subjected to PEG6000-induced osmotic stress. Leaf samples were collected at 0, 4, 8, 16, and 24 h after treatment. Transcript levels were determined by qRT-PCR and normalized to *ZmTublin*. Asterisks indicate significant differences (** $P < 0.01$, Student's t-test). The experiment was performed using three biological replicates.

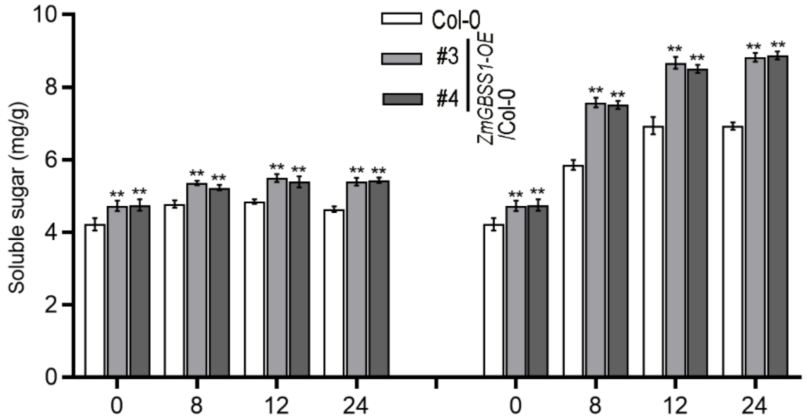

Supplemental Figure S5. The content of soluble sugars induced by PEG6000. Soluble sugar contents following PEG6000-induced osmotic stress at 0, 8, 12, and 24 h. For osmotic stress assays, 20-day-old plants were irrigated with 30% (w/v) PEG6000 solution. Statistical significance was determined using Student's *t*-test (** $P < 0.01$). Data represent mean ± SD (n ⩾ 10).

Supplemental Table S1. Primers used in this study.

Gene (Gene ID)	Primer name	Sequence (5'→3')	Reference
**For *ZmGBSS1* genes cloning and plant transformation**			
ZmGBSS1 (Zm00001eb305810)	ZmGBSS1-F	FCTGGCGGCCGCTCGAGTAATGGCTGCAACGATGG GTTC	
	ZmGBSS1-R	ATTCGAGCTCACTAGTTCACGGAGTGGCTACGTTTT	
**For Subcellular localization**			
ZmGBSS1 (Zm00001eb305810)	GFP-ZmGBSS1-F	ACGGGGGACTCTTGACCATGGCAATGGCTGCAACG ATGGGT	
	GFP-ZmGBSS1-R	AAGTTCTTCTCCTTTACTAGTCGGAGTGGCTACGTT TTC	
**For RT-qPCR**			
ZmGBSS1 (Zm00001eb305810)	ZmGBSS1-QF	CCTCCTCTACTTGAGTGCCG	
	ZmGBSS1-QR	TTCAATGATGACCTCGCGCT	
ZmTublin (Zm00001eb215710)	ZmTublin-QF	ACTTCATGCTTTCGTCCTACGCTCCA	
	ZmTublin-QR	CTGGGAGGCTGGTAGTTGATTC	
AtFT (AT1G65480)	AtFT-QF	AGTCCTAGCAACCCTCACCT	
	AtFT-QR	CCTGCAGTGGGACTTGGATT	
AtSOC1 (AT5G61770)	AtSOC1-QF	TGCATCACTTGCGAAAGCAC	
	AtSOC1-QR	GCTCGCTGTTTTCGATTGCT	
AtActin2 (At3g18780)	AtActin2-QF	GGAATCGTTCACAGAAAATG	
	AtActin2-QR	CAAACAAATGGAGAAGCAAA	
AtP5CS1 (AT2G39800)	AtP5CS1-QF	TTGATGGGAAGGCTTGTGCT	
	AtP5CS1-QR	TTCCAACGCCAGTAGAGCAG	
AtP5CS2 (AT3G55610)	AtP5CS2-QF	GCCTGCACCGTTGAAATTGT	
	AtP5CS2-QR	ATTCCCACCTCAGCACCAAG	
AtBAM1 (AT3G23920)	AtBAM1-QF	ATGTGTGGTGGGGTTTGGTT	
	AtBAM1-QR	GGAGTGTATCAGCGCCAAGT	
AtBAM3 (AT4G17090)	AtBAM3-QF	GGCACTACAACACCAGGTCA	
	AtBAM3-QR	TGTCGCGTTCTGTACTTGCT	
AtDPE1 (AT5G64860)	AtDPE1-QF	GCTGCATTCTCTTCAACCGC	
	AtDPE1-QR	AGCCGTCCGTACAATGACAA	
AtGBSS1 (AT5G64860)	AtGBSS1-QF	AGGTTGTGGGCAAAACAGGA	
	AtGBSS1-QR	CTCAAGAGCAGCCTGACACA	
